# Fatal overdose from injection of human growth hormone; a case report and review of the literature

**DOI:** 10.1186/s12902-022-01193-2

**Published:** 2022-11-08

**Authors:** Azam Erfanifar, Mahsa Mahjani, Sepehr Gohari, Hossein Hassanian-Moghaddam

**Affiliations:** 1 Department of Internal Medicine, School of Medicine, Loghman-Hakim Hospital, Shahid Beheshti University of Medical Sciences, Tehran, Iran; 2 School of Medicine, Shahid Beheshti University of Medical Sciences, Tehran, Iran; 3 Department of Family Medicine, Alborz University of Medical Science, Alborz, Iran; 4 Student Research Center, School of Medicine, Zanjan University of Medical Sciences, Zanjan, Iran; 5 Social Determinants of Health Research Center, Shahid Beheshti University of Medical Sciences, Tehran, Iran; 6 Department of Clinical Toxicology, Loghman-Hakim Hospital, School of Medicine, Shahid Beheshti University of Medical Sciences, South Karegar Street, Kamali St, Tehran, Iran

**Keywords:** Human growth hormone, Somatropin, Abuse, Poisoning, Mortality

## Abstract

**Background:**

Human growth hormone (HGH) is a categorized as a performance-enhancing substance. HGH has been abused by athletes for doping purposes.

**Case presentation:**

We present a first lethal case of HGH acute toxicity. A young-agitated-athlete with a history of somatropin for the past 2-year, who had hallucinations referred to the emergency department reporting to have abused of 300 mg subcutaneous injections of HGH. He was tachycardic with mild hypertension. Lab data revealed hypernatremia (157 mEq/L), hyperkalemia (5.3 mEq/L), high LDH (1448 U/L), and CPK (2620 U/L), in favor of rhabdomyolysis. Routine drug screening tests were negative for all substances. He was intubated due to low O_2_ saturation and progressive loss of consciousness. After several episodes of hyperthermia, hypertension, and possibly pulmonary embolism, he died subsequent to somatropin overdose.

**Conclusions:**

Complications of HGH misuse can be life-threatening and athletes should be warned of its deleterious effects.

**Supplementary Information:**

The online version contains supplementary material available at 10.1186/s12902-022-01193-2.

## Background

Human growth hormone (HGH) or somatropin is a peptide secreted from pituitary gland that stimulates numerous metabolic pathways in cells and plays an essential role in human physiology [1, 2]. It is in the category of Anabolic Agents on the World Anti-Doping Agency (WADA) Prohibited List and is banned at all times and for all categories of athletes. HGH is predominantly mediated by Insulin-like growth factor 1 (IGF-1) that is mainly produced by liver. The advent of recombinant human GH (rHGH) has led to a marked increase in the use of growth hormone (GH) as replacement therapy [2]. Traditionally the drug has implications in the management of pediatric patients with growth hormone deficiency [3]. HGH is stratified in the category of anabolic agents which is commonly used for doping purposes and is prohibited among athletes in sports [2]. Performance-enhancing substances are being used worldwide as they can promote both strength and sprint capacity, however they are accompanied by harmful impact on different organs [4]. Unfortunately, somatropin injections has gained popularity among athletes and are readily available to purchase in many other countries [4–9]. Here, we present a lethal case of somatropin abuse.

## Case presentation

**Fig. 1:**
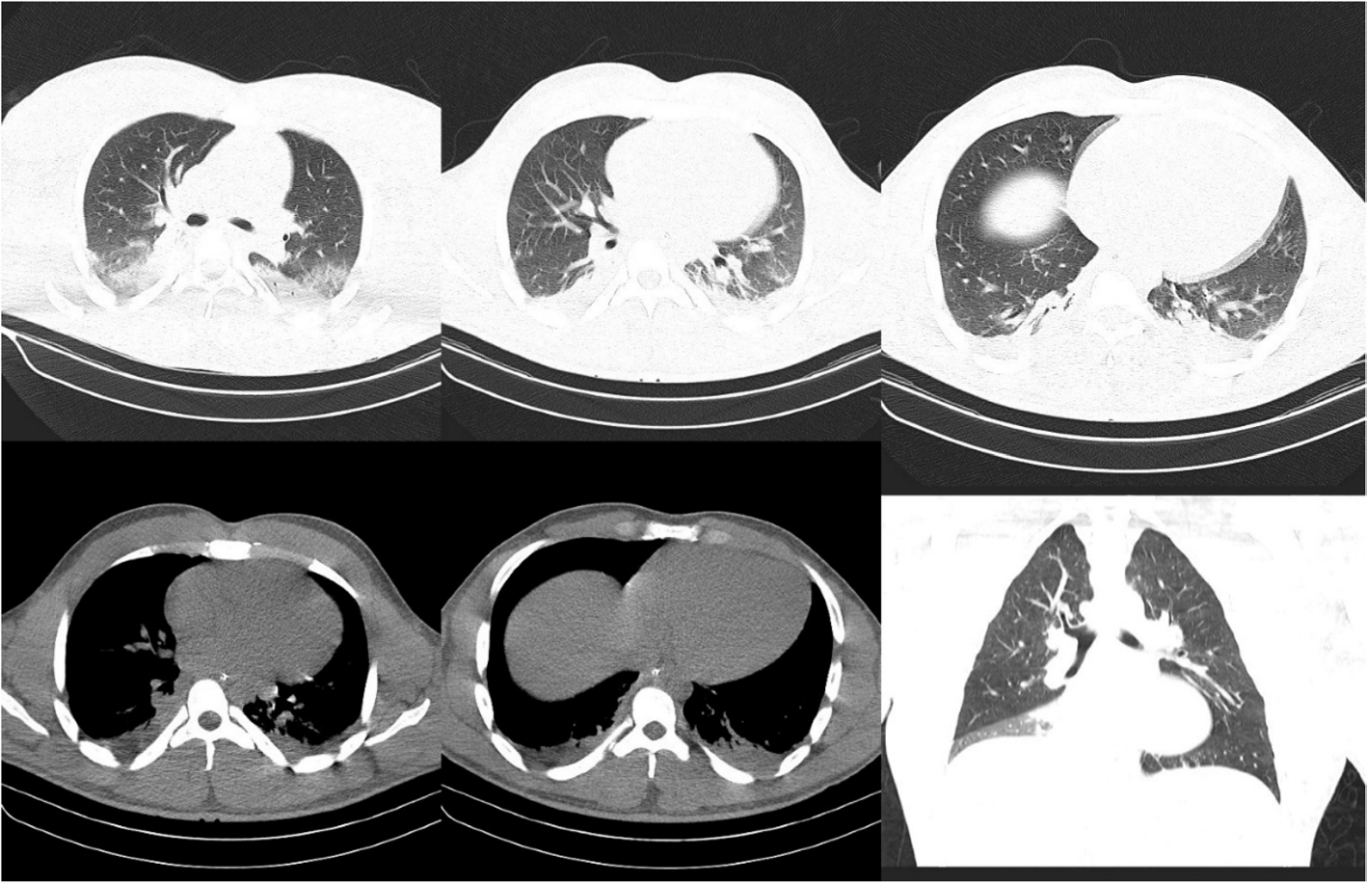
Chest CT scan; consolidations in the dependent areas of lungs are indicative of aspiration pneumonia

An agitated 20-year-old boy without known past medical history, who had hallucinations referred to emergency toxicology department. He was reported to have abused 30 subcutaneous injections of somatropin, each containing 10 mg/1.5 mL all at once with the aim of enhancing performance for coming competition. He also had been using somatropin for the past 2 years for anabolic purposes. The initial vital signs were; BP = 145/96 mmHg, PR = 111/min, RR = 21/min, T = 37.1^0^C and SPO_2_ with mask = 100%. He had been sedated in ED with midazolam. Laboratory tests (Table 1) revealed hypernatremia (157 mEq/L), hyperkalemia (5.3 mEq/L), high Lactate Dehydrogenases (LDH; 1448 U/L) and Creatine Phosphokinase (CPK; 2620 U/L) level which were all robustly signifying that a state of rhabdomyolysis had happened. Urine toxicology analysis was negative for all substances. The supine chest X-Ray had veiling opacities related to pleural effusion. In Electrocardiogram (ECG), left atrium (LA) abnormality was seen according to Romhilt‐Estes criterion (Supplementary figure). The O_2_ saturation level dropped within hours and he had to be intubated. Brain CT scan demonstrated no pathologic changes. Consolidations in dependent sites of lungs were observed in his chest CT scan that were indicative of aspiration pneumonia (Fig. 1). He experienced several episodes of hypertensive crisis during his admission and was then infused on trinitroglycerin drip. On the fourth day he had a temperature of 40 ^0^C. The patient was evaluated for sepsis and samples of endotracheal tube were collected for culture which consisted of gram-positive staphylococcus aureus. Urine culture was also positive for klebsiella. Therefore, antibiotics including; ceftriaxone and clindamycin, were prescribed. As the consciousness level had not been improving up until then, and SPO_2_ levels had begun to decline, the decision was made to change the endotracheal tube and re-intubate him with suspicion of upper airway obstruction. In spite of no ischemic changes in electrocardiography, on the sixth day of admission; elevated troponin level (0.99 ng/ml) was detected. The case was consulted with cardiologists and considering his high d-dimer, they advised chest CT angiography with a high suspicion for pulmonary embolism, however, in view of his unstable condition CT angiography could not have been carried out. As a result, an alternative regimen of anticoagulant therapy with heparin was initiated. The next day he deteriorated with cardiopulmonary arrest, cardio pulmonary resuscitation was instituted but the cardiac rhythm remained unviable and he was declared dead after 40 min.

**Table 1:** Lab tests on admission

		Normal ranges
**White blood cell (/L)**	15,800	4500–11,000
**Hemoglobulin (mg/dl)**	16.4	13.2–16.6
**Platelet (/L)**	455,000	150,000–450,000
**Sodium (mEq/L)**	157	135–145
**Potassium (mEq/L)**	5.3	3.6–5.2
**Fasting blood sugar (mg/dl)**	146	< 100
**Urea (mg/dl)**	33	6–24
**Creatinine (mg/dl)**	1.6	0.7–1.3
**AST (U/L)**	157	10–40
**ALT (U/L)**	359	19–25
**CPK (U/L)**	2620	39–308
**CKMB (U/L)**	374	5–25
**LDH (U/L)**	1448	140–280
**D-Dimer (ng/mL)**	3012	< 0.5
**Lactate (mg/dl)**	10	0.5–1
**Calcium**	9.1	8.6–10.3
**Phosphorus**	3.8	2.8–4.5
**Magnesium**	2	1.7–2.2
**Albumin**	8.9	3.4–5.4
***VBG:***		
**PH**	7.33	7.35–7.45
**HCO**_**3**_ **(mEq/L)**	34.4	23–29
**PCO**_**2**_ **(mmHg)**	66.2	35–45
**PO**_**2**_ **(mmHg)**	122	80–100

## Discussion and conclusion

HGH abuse in sports is widespread due to perceived, though mainly unproven, benefits and the difficulty of detection [4, 7, 9]. Exogenous GH administration, in matured and healthy adults with sufficient GH secretions from pituitary gland, is strongly condemned due to the many adverse effects that can take place in both short-term and long-term use. Doses used by athletes are estimated to an average daily dose of 1–2 mg GH, which is three times higher than the normal endogenous secretions [10]. Current evidence lacks certain recommendations for the overdose level of GH. Acute HGH overdose can cause tremors, drowsiness, dizziness and nausea, although critical acute intoxication in the manner of other drugs like alcohol and opioids have not been reported and has remained unknown. In the long-term setting, excessive administrations of HGH can be a mimicker of acromegaly and its manifestations [6]. GH contributes to collagen and muscle mass increase in heart and leads to cardiomyopathy [11]. Interstitial fibrous tissue proliferation in myocardium is provoked by GH [12]. Such alterations are associated with arrythmias and heart failure [13, 14]. Electrocardiography studies and Holter recordings have documented abnormalities of cardiac rhythm in patients with acromegaly [14]. Left ventricular hypertrophy (LVH) is common in acromegaly patients as a result of volume overload. Since LVH is associated with increased risk for adult sudden death; cardiomegaly secondary to chronic HGH misuse may heightens the risk of sudden cardiac arrest in athletes [15].

Systemic hypertension is promoted via the activation of renin–angiotensin–aldosterone-system (RAAS) advancing oxidative stress as well as sodium and water retention that are stimulated by HGH. Elevated blood pressure have had correlations with the severity of acromegaly cardiopathy [16, 17].

GH impact on neurocognition has been controversial; while GH secretions regulate metabolic and growth function, overexpression of GH in a rodent model caused impairments in memory [18]. Although hallucinations as an aftermath of abundant use of GH are not mentioned as the side effects, few episodes of psychosis in the context of acromegaly were seen [19].

Aspiration pneumonia seen on chest CT scan is probably stemmed from his decreased level of consciousness prior to his intubation. On account of the mere left atrial (LA) abnormality that was seen in the electrocardiograms, and no reflection of acute myocardial infarction, therefore rhabdomyolysis is supposed to be responsible for the elevated troponin level. In our case, acute skeletal muscle hypertrophy and destruction might have resulted in rhabdomyolysis [20]. Hypertensive crisis and the pleural effusion are concluded to be directly ensued from his somatropin toxicity which were supposedly brought about by its fluid retention effect. The high d-dimer could have been suggestive of a probable pulmonary embolism (PE), however in light of his rapid deterioration, chest CT angiography could not be performed. Eventually the cardiovascular collapse is deemed to be attributed to PE. On the other hand, based on his chronic use of HGH, an increased ventricular mass and acromegaly related cardiomyopathy might had been accountable for the sudden cardiac arrest.

As far as we know, this is a first lethal case of HGH acute toxicity considering the amount of abused GH and the extent of severity. Clinicians and in particular; endocrinologists and sport medicine specialists are increasingly confronted with the complications of HGH misuse. Thus, public awareness about its deleterious side effects should be raised.

## Supplementary Information

**Additional file 1: Supplementary Figure 1:** The electrocardiogram of patient. Left atrial abnormality is visible. According to Romhilt-Estes criteria, the second part of P wave deflection in lead V1 represents the duration of ≥ 40 msec and the depth of ≥ 1 mm.

## Data Availability

The data that support the findings of this study are available from the corresponding author upon reasonable request.

## Abbreviations

CT: Computed Tomography
ECG: Electrocardiography
ED: Emergency Department
GH: Growth Hormone
HGH: Human Growth Hormone
LA: Left Atrium
LVH: Left Ventricular Hypertrophy
PE: Pulmonary Embolism
