# Supplementary figures and images for: Fatal overdose from injection of human growth hormone; a case report and review of the literature
Source: BMC Endocr Disord. 2022 Nov 8;22:271. doi: 10.1186/s12902-022-01193-2 (PMC9644502; doi:10.1186/s12902-022-01193-2)

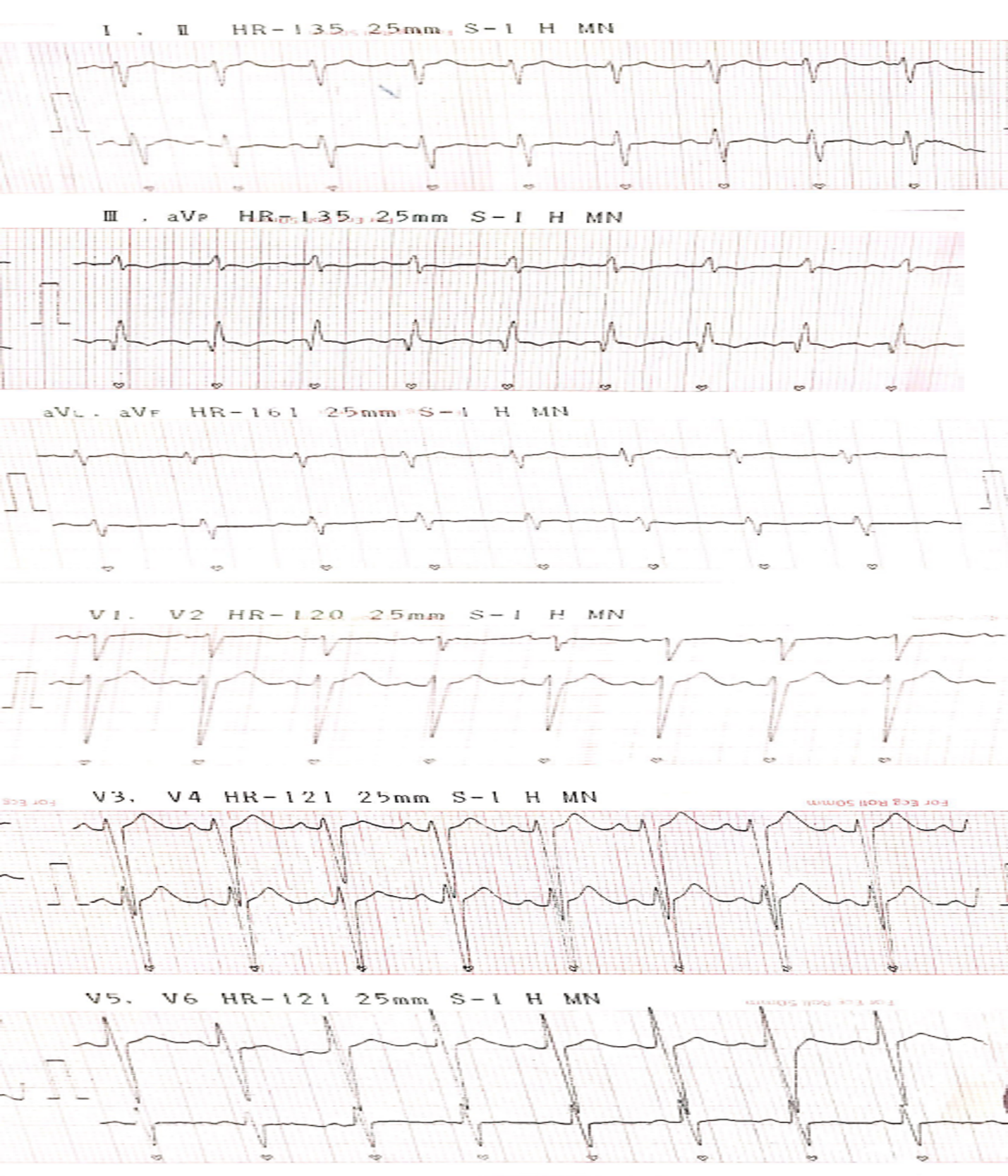

Supplement: Supplementary file 1 — Additional file 1: Supplementary Figure 1: The electrocardiogram of patient. Left atrial abnormality is visible. According to Romhilt-Estes criteria, the second part of P wave deflection in lead V1 represents the duration of ≥ 40 msec and the depth of ≥ 1 mm. [file 12902_2022_1193_MOESM1_ESM.jpeg]
